# Hepatic Metabolomic Responses to Low-Temperature Stress in the Invasive Turtle, *Trachemys scripta elegans*

**DOI:** 10.3390/ani14162388

**Published:** 2024-08-17

**Authors:** Huo-Bin Tang, Qiao-Hong Guo, Jia-Meng Yang, Jin-Hui Zhang, Hong-Liang Lu

**Affiliations:** School of Life and Environmental Sciences, Hangzhou Normal University, Hangzhou 311121, China

**Keywords:** cold exposure, physiological basis, stress response, energy requirement, amino acid metabolism, digestive performance

## Abstract

**Simple Summary:**

To address the physiological response to low-temperature stress in the invasive turtle species, we investigated the hepatic metabolite alteration of *Trachemys scripta elegans* hatchlings under low-temperature treatments. Our results indicated that the levels of hepatic metabolites, e.g., stearolic acid, taurohyocholate, and spermidine, changed significantly in low-temperature-exposed turtles. Despite having only limited impacts on fatty acid and energy metabolism, short-term low-temperature exposure might alter the immune ability of turtles.

**Abstract:**

Investigating the physiological and biochemical changes of ectothermic species before entering hibernation would contribute to the understanding of how they adapt to low-temperature environments. Here, red-eared slider turtle (*Trachemys scripta elegans*) hatchlings were maintained under different thermal treatments (24 °C, slowly decreasing temperatures from 24 °C to 14 °C, and to 4 °C). Hepatic metabolite alterations were measured to assess the metabolic impacts of low-temperature stress in this species. Of these differentially changed metabolites, some (e.g., raffinose, spermidine, allocholic acid, taurohyocholate, 2-ketobutyric acid, acetylcysteine) were shown to decrease, while others (e.g., stearolic acid, D-mannose) increased in low-temperature treatments. Our results indicated that short-term low-temperature stress might have limited impacts on lipid and energy metabolism in this species. The changes in other metabolites (e.g., allocholic acid, taurohyocholate, spermine, acetylcysteine) might be associated with a low food intake (and thus reduced digestive performance) and weakened immune ability of low-temperature-exposed animals.

## 1. Introduction

Environmental temperature has a far-reaching impact on various aspects of organisms [1,2]. In ectothermic species, excessively high or low temperatures hinder their growth and development, and lead to death when falling outside their viable thermal range [2]. Although several behavioral strategies might be employed by animals to mitigate the adverse effects of high- or low-temperature stresses, various physiological changes caused by such stresses would still occur [1]. Animals distributed in high-latitude and/or high-altitude regions would inevitably undergo a certain period of low-temperature stress. Of them, amphibians and reptiles commonly enter into a state of dormancy before the onset of freezing weather (i.e., hibernation), and they can reduce their organ size, metabolic rate, and heart rate, elevate their blood glucose level, and alter their gene expression and metabolite profile to adapt to low-temperature stress during hibernation [3,4,5,6]. Physiological and biochemical changes induced by low-temperature stress have been investigated in some species of fish and amphibians [7,8,9,10,11,12]. However, studies considering these changes under low-temperature stress still remain limited in reptile species [13,14,15,16].

*Trachemys scripta elegans* is a freshwater turtle species naturally distributed in the southern United States and northeastern Mexico, and has successfully invaded many regions of the world, including Africa, Asia, and Europe [17,18]. In the natural environment, *T. scripta elegans* individuals are often active when water temperature is above approximately 22 °C, and their body temperature ranges from 22 to 37 °C. This species exhibits temperature-dependent sex determination, with the pivotal temperature (yielding a 1:1 offspring sex ratio) being around 28–29 °C [19]. *T. scripta elegans* can slow their heart rate and blood flow rate and reduce their energy requirements to cope with low environmental temperatures during winter [5,14,20]. Recently, omics techniques have been applied to explore the physiological and biochemical changes of aquatic organisms under low-temperature stress, and reveal that some biological functional pathways, such as energy metabolism, lipid metabolism, adaptive and innate immune responses, etc., are changed markedly in fish under cold stress [12,21,22,23,24,25]. Transcriptional profiling also showed that the expression of genes related to cell damage repair, innate immunity, etc., would be significantly altered in aquatic turtles under low-temperature stress [16]. However, to date, the molecular basis of adaptation to low-temperature stress in those turtle species, such as *T. scripta elegans*, is still not well understood. In this study, we measured the alterations in hepatic metabolites of *T. scripta elegans* hatchlings after being maintained under different temperature regimes (24 °C, slowly cooled to 14 °C or 4 °C from 24 °C). We aimed to explore physiological and biochemical effects caused by low-temperature stress in this species.

## 2. Materials and Methods

### 2.1. Experimental Animal Collection

We collected fertilized eggs of *T. scripta elegans* from a private hatchery in Huzhou (Zhejiang province, China) and incubated them in containers filled with moist vermiculite (−12 kPa). Containers were placed inside a climate-controlled chamber set at 29 °C (close to the pivotal temperature of *T. scripta elegans* [19]). Most eggs were hatched after approximately 2 months. Hatchling turtles were housed individually in tanks (diameter × height: 20 cm × 30 cm, water depth: about 3 cm), and fed a commercial diet daily a few days later.

### 2.2. Experimental Treatment and Sample Preparation

**Figure 1:**
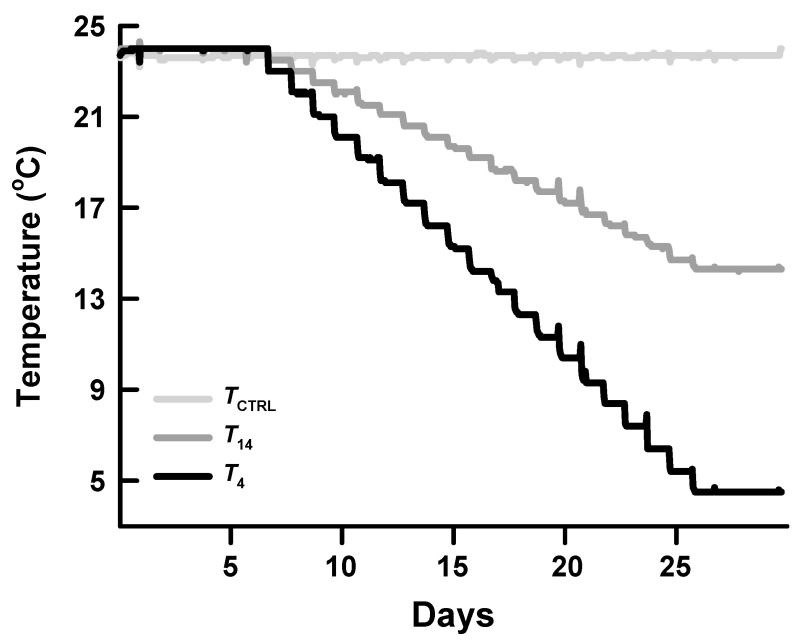
Schematic diagram showing different temperature treatments.

Twenty-four hatchling turtles (mean ± standard error = 9.28 ± 0.27 g) were selected randomly, assigned equally to three treatments, and then placed inside 3 climate-controlled chambers with different temperature conditions but an identical photoperiod of 12 h of light and 12 h of dark (housed in tanks individually). The gender of the hatchling turtles was impossible to identify from appearance; therefore, the gender effect was not considered in this study. In the control group, turtles were maintained in a chamber kept at 24 ± 0.5 °C for 30 days (hereafter referred to as *T*_CTRL_). In the other two groups, the temperatures of chambers were slowly cooled from 24 °C to 14 (hereafter referred to as *T*_14_) or 4 °C (*T*_4_) over a period of 19 days, and then maintained at 14 or 4 °C for 4 days (Figure 1) [15]. The temperature of 24 °C was chosen in this study to represent a temperature at which *T. scripta elegans* individuals are often active during their nesting season, and 14 °C is a temperature at which turtles commonly do not intake any food, while 4 °C is a temperature at which animals should already be in a hibernating state. The treatments of gradually decreased temperatures were applied to simulate the natural progression of water temperature before the arrival of the cold season. After temperature treatment, 6 turtles from each group were selected randomly and then dissected, and their livers were isolated. The liver samples were flash-frozen in liquid nitrogen and stored at −80 °C until liquid chromatography–mass spectrometry (LC-MS) analysis.

### 2.3. Liver Metabolomic Profiling

Accurately weighed 100 mg of liver tissue from each sample was thawed, processed, and used for LC-MS analysis. Detailed sample preparation, instrumental conditions, LC-MS procedures, and data analysis can be referred to in our previous work [26]. Hepatic LC-MS and data analysis was completed by Suzhou PANOMIX Biomedical Tech Co., Ltd. (Suzhou, China).

### 2.4. Data Analysis

One-way analysis of variance (ANOVA) was used to test the difference in body size (mass) among groups. For hepatic metabolomic data, unsupervised principal component analysis (PCA) and supervised partial least squares discriminant analysis (PLS-DA) were used to analyze among-group variations. Metabolite identification was achieved by comparing the information from available databases (MASSBANK, METLIN and HMDB). A non-parametric Kruskal–Wallis test was used to test among-group differences in spectral areas for key identified metabolites.

## 3. Results

**Figure 2:**
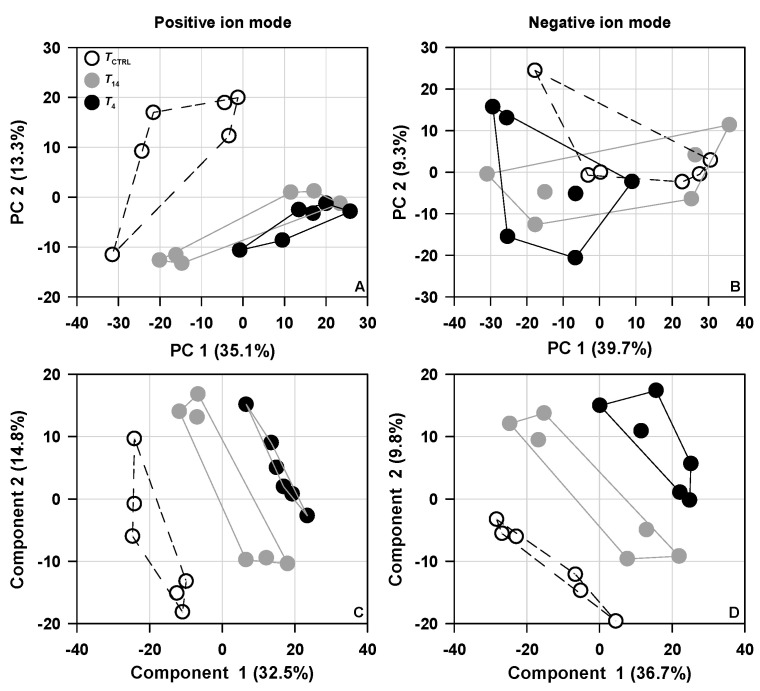
Score plots for principal component analysis [PCA, in the positive (**A**) and negative ion mode (**B**)] and partial least squares discriminant analysis [PLS-DA, in the positive (**C**) and negative ion mode (**D**)] of hepatic metabolite profiles in *Trachemys scripta elegans* hatchlings under different temperature treatments.

**Figure 3:**
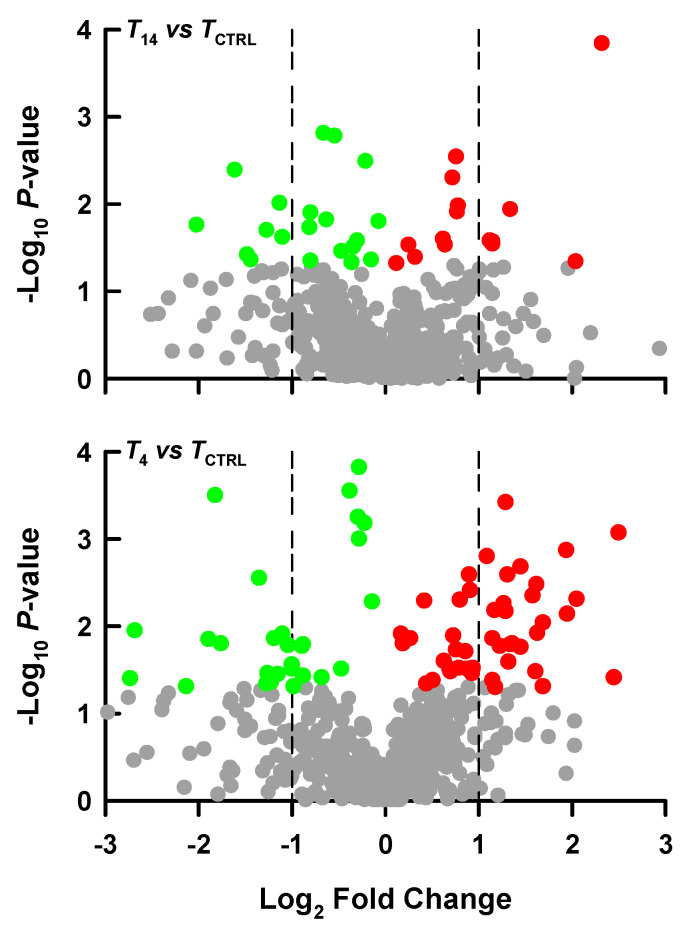
Volcano plots showing hepatic metabolites between the *T*_14_ (slowly cooled from 24 °C to 14) and *T*_CTRL_ (control, 24 °C) groups, and between the *T*_4_ (slowly cooled from 24 °C to 4) and *T*_CTRL_ groups of *Trachemys scripta elegans* hatchlings. In the plots, red and green dots represent significantly increased or decreased metabolites, respectively (*p* < 0.05), while gray dots represent nonsignificantly changed metabolites (*p* > 0.05).

**Figure 4:**
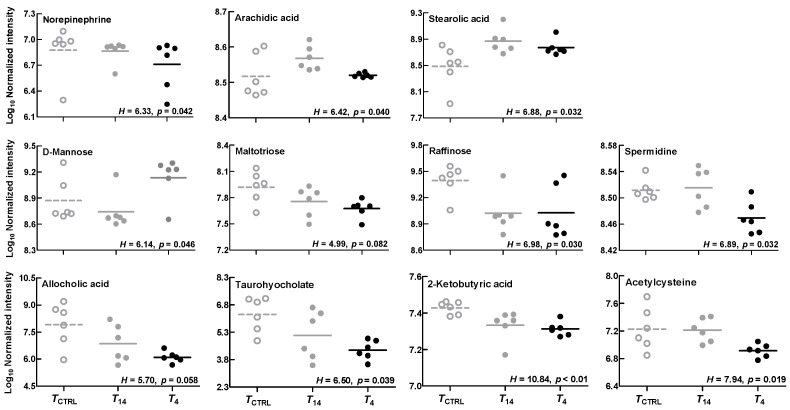
Some differentially changed hepatic metabolites in *Trachemys scripta elegans* hatchlings under different temperature treatments. Non-parametric Kruskal–Wallis tests were used to compare average spectral areas of metabolites.

No significant among-group differences in turtle body size (mass) were found before (*F*_2,21_ = 0.76, *p* = 0.478) and after temperature treatment (*F*_2,21_ = 0.29, *p* = 0.749). For liver metabolomic data, varying degrees of separations among groups could be revealed by multivariate statistical analyses. More than 40% of the total variation could be explained by the first two components given by the principal component analysis (PCA) or partial least squares discriminant analysis (PLS-DA). A more obvious among-group separation degree could be revealed by the PLS-DA analysis than PCA (Figure 2). Compared with the *T*_CTRL_ group, some hepatic metabolites were differentially changed in the *T*_14_ and *T*_4_ groups (Figure 3). The levels of some identified metabolites (e.g., norepinephrine, maltotriose, raffinose, spermidine, allocholic acid, taurohyocholate, 2-ketobutyric acid, acetylcysteine) decreased, while others (e.g., stearolic acid, D-mannose) increased in lower-temperature treatment groups (Figure 4).

## 4. Discussion

Hepatic metabolite changes were measured in *T. scripta elegans* hatchlings maintained under various thermal treatments of slowly decreasing temperatures from 24 °C to 14 °C or 4 °C, and evident metabolic effects of low-temperature stress could be revealed in this study. Transcriptomic responses to low-temperature stress have been evaluated in some mollusk, crustacean, fish, and reptile species [12,16,24,25,27,28]. However, the available study cases considering metabolomic responses to low-temperature stress (or during hibernation) in aquatic organisms are still limited [4,22,29,30].

Multivariate statistical analyses showed some degree of among-group differences in hepatic metabolomic profiles, indicating evident metabolic changes in *T. scripta elegans* hatchlings under low-temperature stress. Considering those identified metabolites, the levels of hepatic amino acids did not change significantly under low-temperature stress (Table S1). Similarly, no or only a few amino acids were shown to alter in fish under thermal stresses based on metabolomic analysis [22,30]. Accordingly, we inferred that potential perturbations of low-temperature stress on amino acid metabolism might be minor in *T. scripta elegans*, as well as in some fish species. Certainly, differential outcomes could be observed across studies considering low-temperature effects on different species. For example, previous transcriptomic studies showed that the pathway of amino acid metabolism would be perturbed in low-temperature-exposed shellfish or fish [12,27]. Furthermore, the levels of some amino acids were shown to decrease significantly in the livers or colonic contents of hibernating frogs [4,29]. It is not surprising to yield discrepant outcomes on amino acid alteration from these studies on various organisms from mollusk to reptile, because they are adapted to different environmental temperatures [12,27,29,30]. As a semi-aquatic turtle species, *T. scripta elegans* individuals are active across aquatic and terrestrial habitats and can withstand a wide range of environmental temperatures [17]. Therefore, we speculated that, compared with other aquatic species (e.g., shellfish, fish, and frogs), *T. scripta elegans* might have a relatively lower sensitivity for metabolic responses, probably because it undergoes greater environmental temperature variations normally.

Stored lipids are an important energy source for animals during fasting or hibernation [8]. Lipid and fatty acid metabolism would be perturbed commonly under low-temperature stress or during hibernation [12,22,27]. Consistent with this prediction, the levels of some fatty acids (e.g., stearolic acid, arachidic acid) were shown to increase in the livers of low-temperature-exposed turtles. Moreover, it is also predicted to enhance the levels of unsaturated fatty acids for maintaining membrane fluidity in fish and other aquatic ectothermic vertebrates under low-temperature stress [30,31,32]. However, no apparent changes in hepatic unsaturated fatty acid levels were found in this study. Similarly, short-term low-temperature exposure seemed to have limited impact on the energy metabolism, although varying degrees of differences in some saccharide levels (e.g., D-mannose, maltotriose, raffinose) were shown here. Certainly, minor modifications in energy metabolism in cold-exposed turtles could be reflected by the changes in several metabolites. For example, 2-ketobutyric acid can be converted to acetyl coenzyme A, thereby participating in the tricarboxylic acid cycle and influencing energy metabolism. Overall, these adjustments of energy metabolism (including a gradual shift from aerobic to anaerobic carbohydrate metabolism, from lipid to carbohydrate catabolism) might occur in animals after long-term cold acclimation or entering hibernation [30,33]. Short-term low-temperature treatment might not have led to the occurrence of these metabolic adjustments. Other strategies (e.g., to alter the respiratory metabolic rate) might be adopted by ectothermic species in response to low-temperature exposure [34]. Bile acids play important roles in many physiological processes, including lipid digestion and absorption, carbohydrate metabolism, and so on [35]. Allocholic acid or some bile acid derivatives (e.g., taurohyocholate) can be the major component in the bile of lower vertebrates, such as fish and reptiles [36]. In this study, reduced hepatic allocholic acid and taurohyocholate levels might be linked with low or no food intake under low-temperature stress and influence the bile composition and digestive performance of turtles. Some metabolites (e.g., spermine and acetylcysteine) have been documented to have anti-inflammatory activity and anti-oxidative stress effects [37,38]. The changes in these metabolites were potentially associated with decreased immune ability of animals under low temperature stress. Therefore, low-temperature stress might have a persistent impact on animal health, which should be investigated further over a long-term period.

## 5. Conclusions

Significant changes in some hepatic metabolites were observed in *T. scripta elegans* hatchlings that were maintained under different low-temperature treatments. Compared with other studies conducted on low-temperature-exposed fish or hibernating frogs [4,22,29,30], the impacts of low-temperature stress on *T. scripta elegans* hatchlings were relatively minor. The changes in some metabolites for bile components might be adaptive responses to low or no food intake under low-temperature stress. Only a few differentially changed metabolites related to energy metabolism pathways could be identified, probably indicating a limited impact of short-term low-temperature stress on energy metabolism in this species.

## Data Availability

All data generated by this study are available in this manuscript and the accompanying Supplementary Materials.

## Supplementary Materials

The following supporting information can be downloaded at https://www.mdpi.com/article/10.3390/ani14162388/s1: Table S1: Spectral areas of some hepatic metabolites in *Trachemys scripta elegans* hatchlings under different temperature treatments. Data are expressed as mean ± standard error.
