# Supplementary material for: Hepatic Metabolomic Responses to Low-Temperature Stress in the Invasive Turtle, Trachemys scripta elegans
Source: Animals (Basel). 2024 Aug 17;14(16):2388. doi: 10.3390/ani14162388 (PMC11350803; doi:10.3390/ani14162388)
Supplement: Supplementary file 1 [file animals-14-02388-s001.zip › animals-3070089-supplementary.pdf]

Supplementary Information:

Table S1. Spectral areas of some hepatic metabolites in *Trachemys scripta elegans* hatchlings under different temperature treatments. Data were expressed as mean ± standard error.

Metabolites	Log_{10} Normalized intensity			Kruskal-Wallis test
	T_{CTRL}	T_{14}	T_4	
L-Valine	9.82 ± 0.05	9.85 ± 0.12	9.55 ± 0.14	$H = 2.33$, $P = 0.312$
L-Leucine	7.61 ± 0.04	7.60 ± 0.03	7.67 ± 0.03	$H = 1.37$, $P = 0.505$
L-Isoleucine	7.42 ± 0.06	7.50 ± 0.07	7.44 ± 0.06	$H = 0.25$, $P = 0.884$
L-Proline	9.49 ± 0.11	9.57 ± 0.02	9.58 ± 0.01	$H = 0.15$, $P = 0.927$
L-Lysine	8.06 ± 0.03	8.04 ± 0.07	8.17 ± 0.10	$H = 0.57$, $P = 0.751$
L-Serine	7.78 ± 0.13	7.93 ± 0.15	7.57 ± 0.08	$H = 4.08$, $P = 0.130$
L-Asparagine	6.90 ± 0.20	7.07 ± 0.20	6.67 ± 0.17	$H = 2.21$, $P = 0.331$
L-Aspartic acid	7.90 ± 0.14	7.64 ± 0.11	7.53 ± 0.19	$H = 4.71$, $P = 0.095$
L-Arginine	6.64 ± 0.06	6.60 ± 0.14	6.71 ± 0.13	$H = 0.50$, $P = 0.778$
L-Threonine	8.64 ± 0.01	8.74 ± 0.19	8.45 ± 0.12	$H = 2.99$, $P = 0.224$
L-Cystine	7.38 ± 0.06	7.22 ± 0.09	7.18 ± 0.08	$H = 2.61$, $P = 0.271$
L-Cysteine	7.01 ± 0.02	7.04 ± 0.02	7.01 ± 0.01	$H = 1.82$, $P = 0.402$
L-Histidine	8.38 ± 0.03	8.28 ± 0.03	8.39 ± 0.07	$H = 3.40$, $P = 0.182$
L-Tyrosine	8.60 ± 0.02	8.66 ± 0.12	8.47 ± 0.06	$H = 2.56$, $P = 0.278$
L-Tryptophan	7.57 ± 0.14	7.67 ± 0.13	7.60 ± 0.18	$H = 0.33$, $P = 0.849$
L-Methionine	9.44 ± 0.09	9.38 ± 0.07	9.41 ± 0.08	$H = 0.33$, $P = 0.849$
L-Phenylalanine	10.01 ± 0.17	10.28 ± 0.22	10.18 ± 0.14	$H = 1.20$, $P = 0.548$
L-Glutamine	8.98 ± 0.02	8.99 ± 0.08	9.09 ± 0.10	$H = 0.32$, $P = 0.854$
L-Glutamate	7.74 ± 0.08	7.77 ± 0.01	7.85 ± 0.04	$H = 2.26$, $P = 0.324$
Creatinine	7.54 ± 0.17	7.95 ± 0.10	7.92 ± 0.16	$H = 4.43$, $P = 0.109$
Creatine	10.37 ± 0.07	10.33 ± 0.06	10.30 ± 0.05	$H = 0.57$, $P = 0.751$
Aminoadipic acid	6.99 ± 0.41	5.90 ± 0.25	6.71 ± 0.38	$H = 3.40$, $P = 0.182$
L-Malic acid	5.79 ± 0.12	5.61 ± 0.08	5.70 ± 0.16	$H = 1.13$, $P = 0.567$
Fumaric acid	8.89 ± 0.07	8.77 ± 0.03	8.91 ± 0.04	$H = 3.89$, $P = 0.143$

Succinic acid	7.40 ± 0.09	7.34 ± 0.07	7.37 ± 0.08	H = 0.29, P = 0.864
ADP	7.64 ± 0.08	7.59 ± 0.07	7.55 ± 0.05	H = 1.06, P = 0.587
NAD	6.82 ± 0.27	7.17 ± 0.10	6.95 ± 0.21	H = 0.78, P = 0.676
NADP	6.44 ± 0.11	6.31 ± 0.09	6.51 ± 0.11	H = 1.72, P = 0.423
NADH	6.77 ± 0.15	7.02 ± 0.21	7.05 ± 0.11	H = 2.01, P = 0.366
Niacinamide	10.40 ± 0.01	10.51 ± 0.08	10.43 ± 0.02	H = 4.67, P = 0.097
D-Glucose	6.89 ± 0.16	7.03 ± 0.09	7.20 ± 0.11	H = 3.56, P = 0.169
D-Fructose	8.75 ± 0.06	8.46 ± 0.13	8.34 ± 0.12	H = 4.99, P = 0.082
D-Ribose	9.24 ± 0.11	9.36 ± 0.13	9.17 ± 0.08	H = 1.45, P = 0.484
D-Arabinose	8.94 ± 0.18	8.92 ± 0.16	9.03 ± 0.14	H = 0.50, P = 0.778
Uracil	8.59 ± 0.07	8.65 ± 0.02	8.54 ± 0.03	H = 2.63, P = 0.268
Glutathione	8.82 ± 0.22	8.56 ± 0.18	8.76 ± 0.11	H = 1.26, P = 0.532
Cholesterol	7.23 ± 0.10	7.43 ± 0.09	7.42 ± 0.15	H = 1.63, P = 0.444
Choline	7.90 ± 0.21	7.81 ± 0.12	7.95 ± 0.19	H = 0.43, P = 0.805
Stearic acid	7.86 ± 0.13	7.99 ± 0.09	8.10 ± 0.05	H = 4.53, P = 0.104
Stearidonic acid	7.40 ± 0.11	7.65 ± 0.21	7.48 ± 0.18	H = 2.26, P = 0.324
Myristoleic acid	6.94 ± 0.03	6.97 ± 0.02	7.01 ± 0.04	H = 2.47, P = 0.291
Linoleic acid	8.57 ± 0.04	8.60 ± 0.07	8.68 ± 0.09	H = 0.61, P = 0.738
Myristic acid	6.96 ± 0.14	6.86 ± 0.15	6.99 ± 0.11	H = 2.54, P = 0.281
Uridine	7.01 ± 0.25	6.62 ± 0.06	6.77 ± 0.12	H = 1.45, P = 0.484
Dopamine	9.71 ± 0.22	9.90 ± 0.13	9.71 ± 0.11	H = 0.67, P = 0.717
γ-Aminobutyric acid	9.71 ± 0.13	9.89 ± 0.12	10.06 ± 0.21	H = 2.54, P = 0.281
Indole	7.49 ± 0.03	7.46 ± 0.01	7.49 ± 0.02	H = 0.71, P = 0.700
L-Methionine S-oxide	7.58 ± 0.14	7.69 ± 0.11	7.80 ± 0.11	H = 1.56, P = 0.459
Inosine	5.94 ± 0.09	6.11 ± 0.04	6.00 ± 0.05	H = 4.67, P = 0.097
IMP	8.88 ± 0.06	9.12 ± 0.16	8.79 ± 0.12	H = 1.82, P = 0.402
myo-Inositol	6.46 ± 0.18	6.41 ± 0.13	6.12 ± 0.08	H = 2.85, P = 0.240
